# Supplementary material for: Gender-Specific Effect of Couple-Based Intervention on Behavioral and Psychological Outcomes of Older Adults with Type 2 Diabetes during the COVID-19 Partial Lockdown in Guangzhou, China
Source: Healthcare (Basel). 2022 Nov 15;10(11):2290. doi: 10.3390/healthcare10112290 (PMC9690585; doi:10.3390/healthcare10112290)
Supplement: Supplementary file 1 [file healthcare-10-02290-s001.zip › healthcare-1991541-supplementary.pdf]

Table S1 Gender-specific differences on outcomes between intervention groups by distance to the high-risk areas of COVID-19 at the COVID-19 survey (n=156) ǂ

	**Lifestyle-related behavior change**		**Self-care activities**		**Depression**	
	β	CI	*β*	CI	*β*	CI
Distance	0.06	(-0.07, 0.19)	0.11	(-0.66, 0.88)	-0.24	(-0.46, -0.02) *
Male	1.53	(0.24, 2.81) *	2.15	(-5.39, 9.7)	-3.43	(-5.62, -1.24) **
Intervention	1.31	(0.03, 2.59) *	0.5	(-7.09, 8.14)	-2.05	(-4.25, 0.16)
Male × distance	-0.01	(-0.37, 0.34)	-0.79	(-2.96, 1.46)	0.53	(-0.07, 1.14)
Intervention × distance	0.09	(-0.29, 0.47)	0.46	(-1.88, 2.86)	0.37	(-0.34, 1.08)
Male × intervention	-1.81	(-3.69, 0.05)	-1.07	(-11.97, 9.78)	1.6	(-1.56, 4.75)
Male × intervention × distance	0.08	(-0.48, 0.65)	-0.74	(-4.1, 2.62)	-0.78	(-1.77, 0.22)

ǂ Adjusted for age, sex, education, and retirement status.

*$P < 0.05$.

**$P < 0.01$.

Table S2 Longitudinal changes of gender-specific difference on outcomes between intervention groups by distance to the high-risk areas of COVID-19 (n=156) [ǂ]

	Self-care activities		Depression	
	β	CI	*β*	CI
Distance	-0.24	(-0.74, 0.26)	-0.13	(-0.34, 0.09)
Male	0.77	(-4.68, 6.21)	-1.93	(-4.00, 0.15)
Intervention	-2.73	(-8.37, 2.91)	1.14	(-0.99, 3.27)
Time [a]				
Time (three-month)	2.48	(-2.43, 7.39)	/	/
Time (COVID-19)	4.00	(-0.63, 8.63)	0.03	(-1.54, 1.59)
Distance × Male	/	/	0.04	(-0.52, 0.61)
Distance × Intervention	/	/	-0.51	(-1.17, 0.16)
Male × Intervention	2.15	(-5.57, 9.86)	-2.26	(-5.34, 0.82)
Distance × Time (COVID-19)	/	/	-0.13	(-0.40, 0.14)
Male × Time (three-month)	5.71	(-1.36, 12.78)	/	/
Male × Time (COVID-19)	-1.67	(-8.25, 4.92)	-1.19	(-3.83, 1.45)
Intervention × Time (three-month)	5.01	(-2.16, 12.18)	/	/
Intervention × Time (COVID-19)	4.09	(-2.85, 11.04)	-2.87	(-5.59, -0.16) *

Distance × Male × Intervention	/	/	0.77	(-0.20, 1.73)
Distance × Male × Time (COVID-19)	/	/	0.48	(-0.24, 1.20)
Distance × Intervention × Time (COVID-19)	/	/	0.76	(-0.09, 1.61)
Male × Intervention × Time (three-month)	-11.68	(-21.69, -1.66) *	/	/
Male × Intervention × Time (COVID-19)	-4.49	(-13.94, 4.95)	3.55	(-0.38, 7.47)
Distance × Male × Intervention × Time (COVID-19)	/	/	-1.42	(-2.66, -0.18) *

ǂ Adjusted for age, sex, education, and retirement status.

[a] Reference: time at baseline.

*$P < 0.05$.
